# Human Parvovirus B19 Induced Apoptotic Bodies Contain Altered Self-Antigens that are Phagocytosed by Antigen Presenting Cells

**DOI:** 10.1371/journal.pone.0067179

**Published:** 2013-06-12

**Authors:** Kanoktip Thammasri, Sanna Rauhamäki, Liping Wang, Artemis Filippou, Violetta Kivovich, Varpu Marjomäki, Stanley J. Naides, Leona Gilbert

**Affiliations:** 1 Department of Biological and Environmental Sciences and Nanoscience Center, University of Jyväskylä, Jyväskylä, Finland; 2 Pennsylvania State College of Medicine/Milton S. Hershey Medical Center, Hershey, Pennsylvania, United States of America; 3 Quest Diagnostics Nichols Institute, San Juan Capistrano, California, United States of America; INSERM-Université Paris-Sud, France

## Abstract

Human parvovirus B19 (B19V) from the *erythrovirus* genus is known to be a pathogenic virus in humans. Prevalence of B19V infection has been reported worldwide in all seasons, with a high incidence in the spring. B19V is responsible for erythema infectiosum (fifth disease) commonly seen in children. Its other clinical presentations include arthralgia, arthritis, transient aplastic crisis, chronic anemia, congenital anemia, and hydrops fetalis. In addition, B19V infection has been reported to trigger autoimmune diseases such as systemic lupus erythematosus and rheumatoid arthritis. However, the mechanisms of B19V participation in autoimmunity are not fully understood. B19V induced chronic disease and persistent infection suggests B19V can serve as a model for viral host interactions and the role of viruses in the pathogenesis of autoimmune diseases. Here we investigate the involvement of B19V in the breakdown of immune tolerance. Previously, we demonstrated that the non-structural protein 1 (NS 1) of B19V induces apoptosis in non-permissive cells lines and that this protein can cleave host DNA as well as form NS1-DNA adducts. Here we provide evidence that through programmed cell death, apoptotic bodies (ApoBods) are generated by B19V NS1 expression in a non-permissive cell line. Characterization of purified ApoBods identified potential self-antigens within them. In particular, signature self-antigens such as Smith, ApoH, DNA, histone H4 and phosphatidylserine associated with autoimmunity were present in these ApoBods. In addition, when purified ApoBods were introduced to differentiated macrophages, recognition, engulfment and uptake occurred. This suggests that B19V can produce a source of self-antigens for immune cell processing. The results support our hypothesis that B19V NS1-DNA adducts, and nucleosomal and lysosomal antigens present in ApoBods created in non-permissive cell lines, are a source of self-antigens.

## Introduction

Human parvovirus B19 (B19V) is a member of the genus *Erythrovirus* of the family *Parvoviridae*. It is a small (20-25 nm in diameter) [1–3] non-enveloped single-stranded linear DNA virus that was discovered in 1975 by Yvonne Cossart [2]. This virus has an icosahedral symmetrical capsid consisting of two structural proteins, viral protein 1 and 2 (VP1 and VP2). The minor capsid protein, VP1, has phospholipase activity that is necessary for viral attachment and cell entry [4,5]. The major capsid protein (95% of the total), VP2, can self-assemble into empty capsids, known as virus-like particles (VLPs). In addition to the capsid proteins, there are three non-structural (NS) proteins, two without known functions, but one known as the cytotoxic NS1 protein. NS1 protein, a member of superfamily 3 of viral helicases, is a pleiotropic nuclear phosphoprotein and absolutely required for viral replication. It is a multi-functional protein that has a role in control of cellular transcription, virus replication, induction of cell death, and transactivation of cellular promoters [6–8].

Erythroid precursors have been shown to be the demonstrated cell type to best support an B19V productive infection [9,10], but other bone marrow hematopoietic lineages support B19V productive infection, although less efficiently [11,12]. B19V NS1 expression plasmids were also used in permissive cells lines to investigate NS1 protein induced cellular apoptosis, cell life cycle, activation of caspase, and DNA fragmentation [13,14]. Other studies have confirmed that B19 virus does infect primary hepatocytes as well as HepG2 cell lines, and that NS1 protein is expressed while promoting apoptosis [15]. Again, B19V NS1 expression plasmids were constructed to determine the involvement of NS1 protein and apoptosis as well as caspase activation [16] in this non-permissive cell line. A transfection efficiency of less then 10% was obtained from this method. In order to increase the expression level of NS1 protein in HepG2 cells, the recombinant baculovirus expression vector system (BEVS) was created to house the NS1 construct [17,18]. Transduction efficiencies of greater than 60% can be obtained with the BEVS system and in this system NS1 expression induced apoptosis and host DNA damage. HepG2 cells were used in the current study because they allow virus binding and internalization, but they are non-permissive for human parvovirus replication [19].

Clinical diagnosis of B19V infection is very common and has been reported worldwide in all seasons [20,21]. The rate of B19V seropositivity rises with age because of continuous exposure to the virus [22]. Clinical features of B19V infection depend on the host’s condition, but common symptoms and signs consist of mild illness with pyrexia, malaise, myalgia, rash, or arthralgia [23–25]. The common diseases caused by B19V infection include erythema infectiosum (fifth disease or slapped cheek syndrome), arthralgia, arthritis, transient aplastic crisis, chronic anemia, congenital anemia, and hydrops fetalis [4,26,27]. These general manifestations of B19V infection are very similar to features of several autoimmune diseases such as acute, chronic and autoimmune hepatitis, as well as acute fulminant liver failure [28–40], systemic Lupus erythematosus (SLE) and rheumatoid arthritis (RA) [26,27,41]. Moreover, this infection has been reported to trigger SLE [41] and RA [42], but the mechanisms are still not fully understood.

It has been shown that B19 viral infection may induce anti-phospholipid antibodies through phospholipase-A2-like activity [43–46] and molecular mimicry mechanism leading to immunological cross reaction [45,47]. Others have suggested that the NS1 protein promotes chronic inflammation by transactivation of cellular promoters for the expression of TNF-α and IL-6 genes [8,48]. It also has been implied that the NS1 protein may be a super-antigenic stimulator for T and/or B lymphocytes [47]. Our previous studies have shown that host cell DNA damage occurs with NS1 expression [16–18,49] and that host DNA is covalently attached to the NS1 protein to form bulky adducts [18,49].

We have proposed that B19V induction of autoimmunity may occur when T cells specific for NS1 protein are stimulated during a reoccurrence of B19V infection or re-stimulation of NS1 expression. NS1 protein specific T lymphocyte helper cells would then provide second signal to anergized autoantigen specific B lymphocytes. We hypothesized that B19V-induced ApoBods contain NS1-modified host cell DNA as well as nucleosomal self-proteins and that NS1 protein in ApoBods would be processed by professional antigen presenting cells such as dendritic cells or macrophages. Simultaneously, anergic anti-dsDNA specific B lymphocyte would take up NS1 protein modified self-DNA through its DNA surface IgM receptor; processing NS1 protein to the B lymphocyte surface would allow NS1 specific T helper cell second signaling. Similarly, anergized B lymphocytes specific for DNA binding proteins or NS1 interacting proteins would take up NS1 as part of DNA-protein complexes. The object of this study was to identify cell constituents associated with B19V-induced ApoBods that could be candidate autoantigens in SLE. Understanding the mechanism by which B19V breaks immune tolerance would argue for detection of initial viral insults and may provide insights leading to better patient treatment strategies.

## Materials and Methods

### Cell Culture

*Spodoptera* *frugiperda* -derived cells (*Sf*9 cells, ATCC-CRL-1711, Manassas, VA), were cultured in spinner flasks using Insect-XPress cell medium (BioWhit-taker®, Walkersville, MD, USA) at 27 °C. Human hepatocellular liver carcinoma cell line HepG2 cells (ATCC-HB-8065), a B19V non-permissive cell line, was cultured in 440 mL of Hepatocyte Minimum Essential Medium (Gibco®, Invitrogen, Carlsbad, CA, USA) supplemented with 50 mL fetal bovine serum (FBS) (Gibco®), 5 mL L-glutamine (Gibco®) and 5 mL Penicillin-Streptomycin (Pen/Strep) (Gibco®). Acute monocytic leukemia derived human monocytes, THP-1 cells (ATCC-TIB-202), were cultured in 440 mL of RPMI-1640 Medium Hybri-Max^TM^ (Gibco®; modified with L-glutamine, 4500 mg/L glucose and 15 mM HEPES; Sigma, Sigma-Aldrich Inc., St. Louise, MO, USA), and supplemented with 50 mL FBS, 5 mL Pen/Strep and 0.05 mM β-mercaptoethanol. Both HepG2 and THP-1 cells were cultured at 37 ^°^C, 5% CO_2_, in tissue culture flasks. For engulfment studies, THP-1 cells were differentiated as previously described by Daigneault and colleagues [50]. In brief, 5 x 10^5^ THP-1 cells were seeded in RPMI medium as described above with addition of 200 nM phorbol-12-myristate-13-acetate (PMA) (Sigma) and incubated for 3 days. Replacing the PMA medium with fresh RPMI-1640 medium supplemented with Pen/Strep and β-mercaptoethanol and incubating the cells for an additional 5 days further enhanced differentiation. Experiments were performed on days 8 to 9 post initiation of differentiation.

### Baculovirus Transduction of HepG2 Cells

Recombinant baculoviruses expressing enhanced green fluorescent protein (*Ac*EGFP) and *Ac*EGFP-NS1 fusion proteins under the CMV immediate-early promoter were prepared using the Bac-to-Bac® Baculovirus Expression system (Invitrogen, CA, USA) as previously reported [17]. The transduction efficiencies (TE) of these viruses were determined by growth of HepG2 0.5 x 10^6^ cells overnight and transduction with recombinant *Ac*EGFP or *Ac*EGFP-NS1. BD FACSCALIBUR flow cytometer (Becton-Dickinson, NJ, USA) was used to verify if the viruses had 70% TE for use in the apoptotic bleb/bodies induction experiments as described by Kivovich and colleagues [17,18]. In the present study, HepG2 cells were transduced with recombinant viruses and kept in the dark rocking at room temperature (RT) for 4 h to promote viral binding to the cellular membrane.

### Induction of Apoptotic Blebs

Induction of apoptotic blebs was performed by seeding 0.5 x 10^6^ HepG2 cells in 6-well culture plates with 4 sterile cover slips per well and incubated overnight at 37 °C in 5% CO_2_. Cells were then transduced at a TE of 70% with recombinant baculoviruses *Ac*EGFP or *Ac*EGFP-NS1. The culture plates were incubated at RT and kept in the dark on a rocking platform for 4 h. Transduced samples were washed once with sterile phosphate buffered saline (PBS), and pre-warmed supplemented medium was added to the culture wells before being incubated at 37 °C in 5% CO_2_.

At 48 h post transduction, supernatant from each well was removed then 2 cover slips were taken to another 6-well plate and fixed with 4% paraformaldehyde (PFA)/PBS for 20 min with rocking at RT. The apoptotic blebs were characterized using a scanning electron microscope (SEM) as described in section 2.4. The remaining 2 cover slips in each well with adherent cell were stained with Annexin V-PE as described in section 2.5.

### Scanning Electron Microscopy (SEM) Imaging

Cover slips with HepG2 cells were finely coated with a conductive gold film layer. The coating was plated with a fine coat sputter machine at a voltage of 0.9-1 kV and a corresponding current of 5-6 mA. The coating time was 4-6 min, leaving a gold layer of approximately 4 nm thick. After coating with gold the samples were examined by SEM.

A scanning electron microscope (SEM; JEOL JSM 820, Tokyo, Japan) equipped with a digital image acquisition card was used to examine samples fixed by screws on a copper specimen holder and transported into the SEM chamber by a sample transfer rod. The SEM accelerating voltage was 3 to 10 kV, and filament current 260 µA. Fields of interest were located at lower magnification, ~ 250 to 350x, and then the height of the stage and focus were adjusted to specify the working distance of 18 mm; image quality was optimized by adjusting X and Y stigmators, brightness and contrast to obtain the best resolution. The image was captured by the SEM Afore program.

### Annexin V-PE Staining

HepG2 cells on cover slips from the previous experiment were submerged in 500 µL Annexin V-PE binding buffer (BioVision, Inc., Milpitas, CA, USA). Each cover slip was incubated with 5 µL Annexin V-PE (BioVision) for 15 min in the dark with rocking at RT. Cells were washed in HBSS (Hank’s Balanced Salt Solution) (Mediatech, Inc, Herndon, VA, USA), supplemented with 3 mM CaCl_2_, and fixed for 15 min in ice-cold 4-6% PFA/HBSS. Stained cover slips were mounted on glass slides with 3-5 µL Prolong Gold Antifade Reagent with DAPI (Invitrogen). The slides were stored at 4 °C before being analyzed by confocal microscope (Olympus).

### Purification of ApoBods

An amount of 10 x 10^6^ HepG2 cells were seeded in 175 cm^3^ cell culture flasks and left to grow at 37 °C in 5% CO_2_ for 24 h. Cells were then transduced with *Ac*EGFP and *Ac*EGFP-NS1 viruses with a TE of 70%. An inducer of cellular apoptosis, staurosporine (a protein kinase inhibitor; S4400 staurosporine from *Streptomyces sp.*, Sigma), was used as a positive control in this study. HepG2 cells seeded simultaneously, using the same seeding density as with the transduced cells, were treated with 1 µM concentration of staurosporine in growth medium in parallel with viral treated samples. At 72 h post transduction, supernatant from each was centrifuged 1,700 x g (Heraeus Labofuge 400, Thermo Fisher Scientific Inc., UK) for 3 min and filtered with gravity through a 5.0 µm filter (Millipore, Billerica, MA, USA).

A volume of 2 mL from the filtered supernatant was taken for verifying the quantity of ApoBods and fluorescence EGFP-NS1 signal by flow cytometry (FC). The supernatant was centrifuged at 16,100 x g for 20 min and the pellet was resuspended in 400 µL Annexin V-PE binding buffer (BioVision). Half of the ApoBods (200 µl) were analyzed directly by FC as described in section 2.7. The other half of the ApoBods were mixed with 10 µL Annexin V-PE (BioVision) and incubated for 5 min in the dark on ice. Samples were centrifuged at 16,100 x g for 20 min and pellets were fixed with 50 µL 4% PFA/PBS at RT for 10 min. Again the samples were centrifuged at 16,100 x g for 10 min and ApoBods pellets mounted with Prolong Gold Antifade Reagent with DAPI (Invitrogen) on a glass slide and covered with a cover slip. Samples were stored at 4 °C and analyzed by wide-field microscopy imaging (Leica DM5500B, GmbH, Germany) (section 2.8).

The remaining filtered supernatant was ultracentrifuged at 285,000 x g (Beckman Coulter Optima L-90K Ultracentrifuge) for 1 h at 4 °C. ApoBod pellets were resuspended in 100 µL PBS overnight at 4 °C. These purified ApoBods were stored at 4 °C for the immunolabeling and engulfment studies (sections 2.9 and 2.10, respectively)

### Flow Cytometry (FC)

FC analysis was performed to determine the amount of apoptotic body induction (section 2.6). Initially the samples were examined for the amount of events within one minute time period with low speed. Then a total of 10,000 events from the filtered ApoBods were examined for the EGFP fluorescent signal with high speed. FC results were measured by the BD FACSCALIBUR flow cytometer (Becton-Dickinson). Data were collected and interpreted by using Cell-Quest pro software version 5.2.1 (Becton-Dickinson). The statistical analysis was performed with FlowJo Flow Cytometry Analysis Software version 8.4.5 (Tree Star Inc., Ashland, OR, USA).

### Wide-field Imaging

ApoBods stained with Annexin V-PE (from section 2.6) were analyzed by Leica fluorescent microscopy (Leica DM5500B) to determine expression of phosphatidylserine (PS) as a consequence of apoptosis. The fluorescence images were taken with a 100x/1.3 oil immersion objective and with excitation filters of 365, 470 and 530 nm. Components of ApoBods were identified by the green fluorescence signal from EGFP or EGFP-NS1, blue signal demonstrating DNA stained with DAPI, and red signal representing PS stained with Annexin V-PE. The appropriate exposure, gain and intensity were adjusted prior to recording the images. ImageJA 1.45B program (National Institutes of Health (NIH), Bethesda, MD, USA) was used to analyze the Leica images.

### Immunolabeling of ApoBods

The purified ApoBods from section 2.6 were immunolabeled for further characterization. A volume of 50 µl ApoBods were pelleted at 16,100 x g for 20 min and fixed with 4% PFA/PBS for 20 min before being washed twice with PBS. The remaining immunolabeling procedure had centrifugation steps (16,100 x g for 20 min) between them in order to remove the supernatant and pellet the ApoBods. Free aldehyde in ApoBods were blocked for 10 min with 0.15% glycine in PBS and permeabilized for 20 min with Triton solution (0.1% Triton X-100 (Fisher, Hampton, NH, USA), 0.01% NaN_3_ and 1% bovine serum albumin (BSA) in PBS). Primary antibodies at a concentration of 1 mg/mL were diluted 1/50 in Triton solution and incubated with the purified ApoBods for 1 h RT while rocking. The specific primary antibodies used were rabbit polyclonal anti-histone H4 (BioVision), mouse monoclonal anti-Ku80 (111, Abcam, Cambridge, UK), sheep polyclonal anti-apolipoprotein-H (ApoH) (Invitrogen, Camarillo, CA), rabbit polyclonal anti-Lamp2 (Abgent, CA, USA), rabbit monoclonal anti-phospho-histone H2B (ser14) (Upstate, NY, USA) or mouse monoclonal anti-Smith (GeneTex, Inc., GmbH, Germany) antibodies. After the incubation, the Apobods were washed in Triton solution for 15 min. The corresponding secondary antibodies (Molecular Probes, CA, USA) used for detection were goat anti-rabbit Alexa Fluor 594 for H4 and H2B, goat anti-mouse Alexa Fluor 633 for Ku80 and Smith, donkey anti-sheep Alexa Fluor 568 for apolipoprotein-H, and goat anti-rabbit Alexa Fluor 633 for Lamp2. The secondary antibodies were diluted at 1/200 in Triton solution and incubated with the ApoBods for 1 h RT while rocking. The ApoBods were washed with Triton solution and then the DNA was stain in 1/1,000 dilution of Hoechst (Sigma) in water for 10 min. Finally, the immunolabeled ApoBods mounted with 10 µL Mowiol^®^ mounting medium (supplemented with 2.5% w/v of Dabco) and covered with a cover slip. Slides were stored at 4 ^°^C until analyzed by confocal microscopy (Olympus).

### Feeding and Labeling THP-1 Cells

For THP-1 immunolabeling, ApoBods collected from 10 x 10^6^ Hep2G cells as described in section 2.6 were suspended into 500 µl of HBSS supplemented with 3 mM CaCl_2_ of which half was used to feed 1 x 10^5^ dTHP-1. The ratio between fed cells and feeder cells used to produce ApoBods were thus 1:100. The ApoBods were added to fresh RPMI-1640 medium, supplemented as described above, and added to cover the dTHP-1. The cells were then incubated for 2 h at 37 ^°^C, 5% CO_2_. After the feeding, the cells were fixed with 4% PFA for 10 min and immunolabeled with rabbit polyclonal anti-Lamp2 (1 mg/mL, Abgent) at a dilution of 1/50 in Triton solution; anti-Lamp2 labeling was detected with 1/200 dilution of goat anti-rabbit Alexa Fluor 594 secondary antibody (Molecular Probes) in Triton solution. As a negative control, phagocytosis was inhibited in THP-1 and dTHP-1 cells by treatment with 1 x 10^-5^ M cytochalasin B (CB) from *Helminthosporiumdematioideum* (Sigma) for 30 min before feeding and maintained throughout the feeding. These immunolabeled samples were stained with Hoescht (Sigma) and mounted as above. Slides were stored at 4 ^°^C until analyzed with laser scanning confocal microscopy (Olympus). Phagocytic activities (PA) of THP-1 (monocytes) and dTHP-1 (macrophages) were analyzed by counting green fluorescence signal inside the cells. Inhibitory effect of CB was also calculated by comparing PA of CB treated and non-treated cells. Percentage PA was analyzed by counting the green signal contained in 1,200 macrophages.

### Confocal Imaging

An Olympus IX-81 with a Fluoview-1000 confocal microscope (Olympus Corporation, Tokyo, Japan) set-up was used for imaging of fixed cells and immunolabeled samples. The images of these samples were taken with 40x and 60x oil immersion objectives at 405, 488 and 543 nm excitation wavelengths; excitation at 633 nm was also used for acquiring images of ApoBods. Induced ApoBods and differentiated macrophages fixed on glass cover slips were identified and examined by using emission filters as follows: 425-455 nm for blue-fluorescence (DAPI), 500-530 nm for green-fluorescence (for Alexa Flour 488), 555-625 nm for red-fluorescence (for Alexa Flour 594), and 625-800 nm purple-fluorescence (for Alexa Flour 633). The appropriate exposure, gain and intensity were corrected before recording the images. These images were analyzed using the ImageJA 1.45B program (NIH).

For ApoBods images, quantification of fluorescence intensity was performed by 3 independent assays of each condition (N = 30) and determined with a free, open source software package, BioImageXD [51]. Levels for the laser power, detector amplification and optical sections were optimized for each channel in confocal microscope before starting the quantification. The volume of the labeled structures from confocal images was evaluated by intensity threshold segmentation. The sum of volumes over the threshold was normalized to average area of the ApoBods. The total area of ApoBods base on the DNA image was quantified using a threshold that distinguished them from the background. Regions for cell area calculation were defined by first smoothing images with Gaussian kernel and thresholding.

### Statistical Analysis

The statistical analyses of the amount of ApoBods, antigen signal in ApoBods, PA and CB inhibition percentages were performed using PASW Statistics software (SPSS Inc., Hong Kong). The calculated means were compared according to the following groups: ApoBods induced by transductions of *Ac*EGFP with *Ac*EGFP-NS1, *Ac*EGFP transduction with staurosporine, and *Ac*EGFP-NS1 transduction with staurosporine. These data were further evaluated by one-way ANOVA post hoc test analysis and expressed as mean ± standard error of the mean (SEM). Values of *P* < 0.05 were considered statistically significant.

## Results

### NS1 protein expression induces production of apoptotic blebs and ApoBods in non-permissive cells

**Figure 1:**
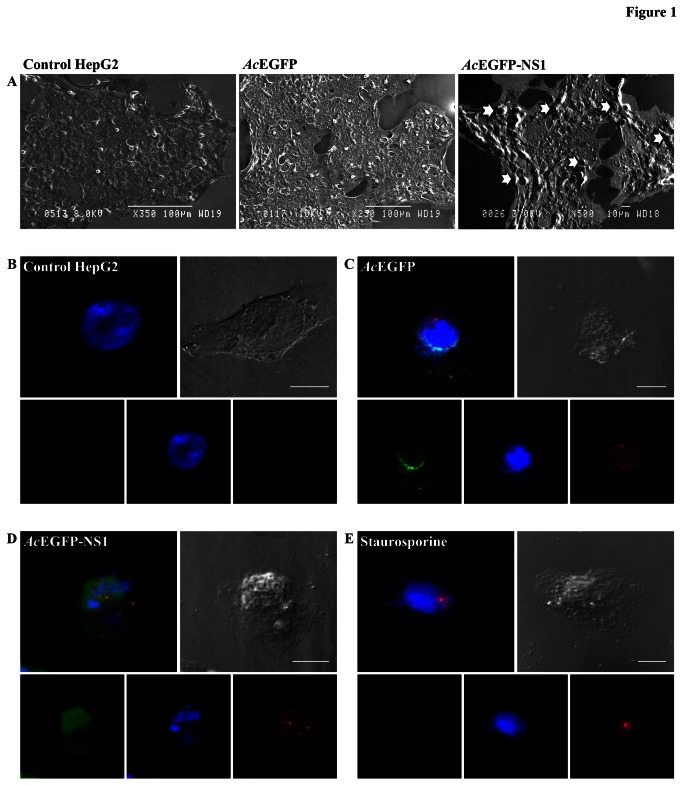
Human Parvovirus B19 NS1 protein induces apoptotic blebs and bodies in non-permissive cell line. (**A**) Scanning electron microscopic images of non-infected cells, cells transduced with *Ac*EGFP and *Ac*EGFP-NS1, respectively. ApoBods with potential self-antigens are detected at 48 h post-transduction as indicated with arrows. Bars 100 µm or 10 µm. (**B**–**E**) Apoptotic blebs created from NS1 expression show positively for Annexin V-PE. Laser scanning confocal microscopy images of (**B**) non-transduced HepG2 cells, (**C**) cells transduced with *Ac*EGFP, (**D**) cells transduced with *Ac*EGFP-NS1, and (**E**) staurosporine treated cells. Cells were visualized (lower panels) directly for EGFP (green), stained for DNA with DAPI (blue), and labeled for phosphatidylserine (PS) with Annexin V-PE (red). Upper panels represent the merged image of the bottom panels and the DIC micrograph to see the morphology of the surface of the cell. Bars 20 µm.

To examine the role of B19V NS1 protein in providing a source of self-antigens characteristic apoptosis events were induced. Apoptotic blebs and hence bodies were created when a non-permissive cell line, HepG2, was transduced with recombinant baculoviruses *Ac*EGFP and *Ac*EGFP-NS1 (Figure 1). Figure 1A illustrates scanning electron micrographs of HepG2 in normal conditions, and then transduced with *Ac*EGFP and *Ac*EGFP-NS1 for 48 h. As displayed in Figure 1A, apoptotic blebbing seen on the cell’s surface (arrows) characteristic of apoptotic events was enhanced with the expression of B19V NS1 protein compared to non-transduced cells or cells transduced with a baculovirus vector expressing EGFP, a protein that does not induce significant apoptosis. Blebbing increased as a consequence of NS1 expression, which was greater than that in *Ac*EGFP transduced or non-transduced cells. When non-transduced (Figure 1B), *Ac*EGFP (Figure 1C) and *Ac*EGFP-NS1 (Figure 1D) tranduced, and staurosporine treated cells (Figure 1E) were viewed directly for EGFP fluorescence (green), DAPI staining (blue) and Annexin V-PE staining (red), cells expressing *Ac*EGFP (Figure 1C) and *Ac*EGFP-NS1 (Figure 1D) displayed Annexin V-PE staining with intensity greater in *Ac*EGFP-NS1 expressing cells compared to *Ac*EGFP. Staurosporine treated cells demonstrated Annexin V-PE staining. DAPI staining demonstrated nuclear fragmentation and apparent blebbing in *Ac*EGFP and *Ac*EGFP-NS1 transduced cells and staurosporine treated cells with destruction more extensive in *Ac*EGFP-NS1 expression cells.

**Figure 2:**
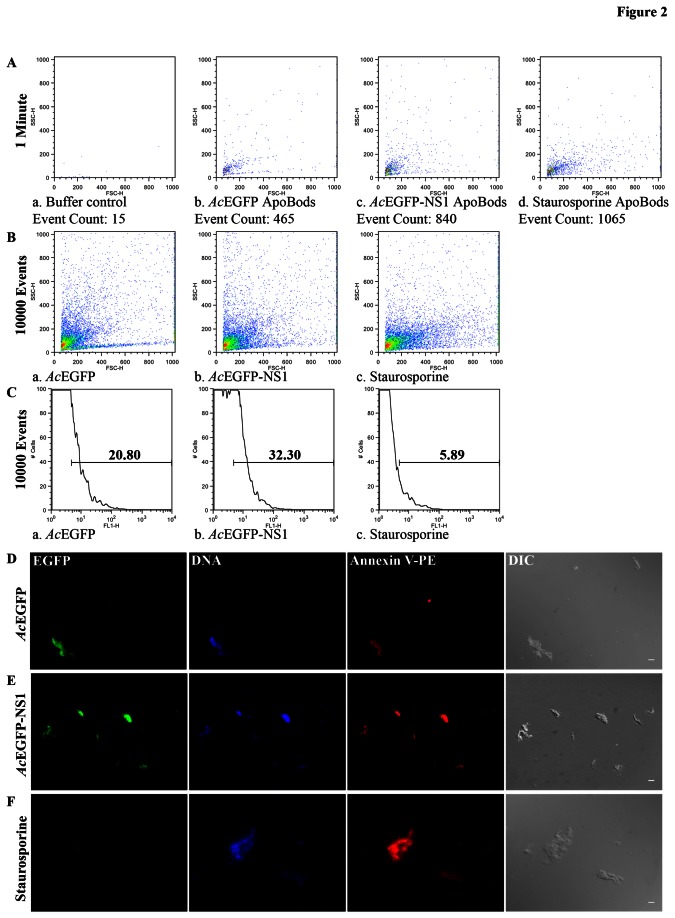
Self-antigens are present in ApoBods created by NS1 expression and those ApoBods can be purified and quantified. Flow cytometry analysis was conducted on purified ApoBods from *Ac*EGFP and *Ac*EGFP-NS1 transduced cells, and staurosporine positive samples. Representative dot plots and histograms are provided. Experiments were conducted independently three times. (**A**) Counts were recorded for 1 min to compare the amount of ApoBods produced in each condition, buffer control, ApoBods produced from transductions of *Ac*EGFP, ApoBods from *Ac*EGFP-NS1 transduction, and ApoBods from staurosporine treated cells. (**B**) Event counts were recorded in order to reach 10,000 events to compare the percentage of green signal from each ApoBods production situation. ApoBods produced from transductions of *Ac*EGFP, ApoBods from *Ac*EGFP-NS1 transduction, and ApoBods from staurosporine treated cells were verified. Buffer control was not added here due to absence of events. (**C**) Histograms of the green fluorescent signal from the previous event counts for each ApoBods production condition. Gated bar indicated the percentage of green signal. Fluorescent microscopy images of purified ApoBods created from (**D**) *Ac*EGFP and (**E**) *Ac*EGFP-NS1 transductions and, (**F**) Staurosporine treated cells. Purified ApoBods were visualized directly for EGFP (green), stained for DNA with DAPI (blue), labeled for PS with Annexin V-PE (red), and visualized with DIC microscopy to see the morphology. Bars 5 µm.

In order to evaluate whether B19V NS1 can generate a large-scale source of ApoBods, transductions of HepG2 cells were performed on a larger scale and ApoBods were consequently purified. After purification, the number of ApoBods was counted by FC (Figure 2A–C). The forward and side scatters from FC studies provided an overview of the apoptotic body population. As shown in Figure 2, dot plot analysis of data was recorded only for 1 min in order to compare the amounts of ApoBods created for each condition; buffer control, *Ac*EGFP transduction, *Ac*EGFP-NS1 transduction and staurosporine treatment as a positive control (Figure 2A–C, respectively). The event counts recorded for each condition showed more ApoBods produced from the transduction of *Ac*EGFP-NS1 (910 ± 70.00) compared to *Ac*EGFP transduction (430 ± 50.74). The number of ApoBods generated by *Ac*EGFP-NS1 transduction was comparable to staurosporine, the positive control of apoptosis, (995 ± 70.00) (Table S1, data not shown). The results demonstrated the ability of NS1 to induce apoptosis in the non-permissive cells, and that ApoBods can be produced in an amount sufficient for further characterization.

Initial characterization of the ApoBods was conducted with the use of FC. 10,000 events counted from the purified ApoBods were displayed in dot plots for *Ac*EGFP and *Ac*EGFP-NS1 transductions, as well as staurosporine treatment (Figure 2B, respectively). The percentage of green signal of ApoBods from *Ac*EGFP (18.06 ± 2.44) or *Ac*EGFP-NS1 (31.77 ± 4.74) transductions calculated from 10,000 events and compared to ApoBods from staurosporine (8.44 ± 1.32) treatment is shown as a histogram in Figure 2C. The approximately time to collect 10,000 events from *Ac*EGFP ApoBods (6.47 ± 0.29 min) were double compared to the time to collect the events from *Ac*EGFP-NS1 (2.57 ± 0.23 min) and staurosporine ApoBods (2.50 ± 0.29 min) (Table S1, data not shown). The analyzed results showed that the percentage of green signal in the ApoBods from *Ac*EGFP-NS1 induction is higher than *Ac*EGFP induction. In addition, these results indicate that during NS1 expression, a high amount of ApoBods can be created from a non-permissive cell line and NS1 remains inside these bodies as seen in Table S1. Figure 2A–C displays a representative experiment from 3 independent assays.

### Nucleosomal and cytosolic self-antigens are in ApoBods induced with B19V NS1 

The presence of EGFP-NS1 in the ApoBods suggests that other associated proteins may also be present, such as DNA damage or associated repair proteins. To further characterize the ApoBods, the bodies were stained with DAPI and Annexin V-PE to see if DNA and PS antigens were present. Representative fluorescent microscopy images of purified ApoBods created from *Ac*EGFP and *Ac*EGFP-NS1 transductions, as well as staurosporine treated cells (Figure 2D–F) indicated the presence of green signal from EGFP and EGFP-NS1 and red signal from Annexin V-PE binding to external membrane PS as seen in Figure 2D and 2E, respectively. Staurosporine induced ApoBods stained for DNA (blue) and PS (red) (Figure 2F). The morphology of the ApoBods were as expected and are shown in the DIC images.

**Figure 3:**
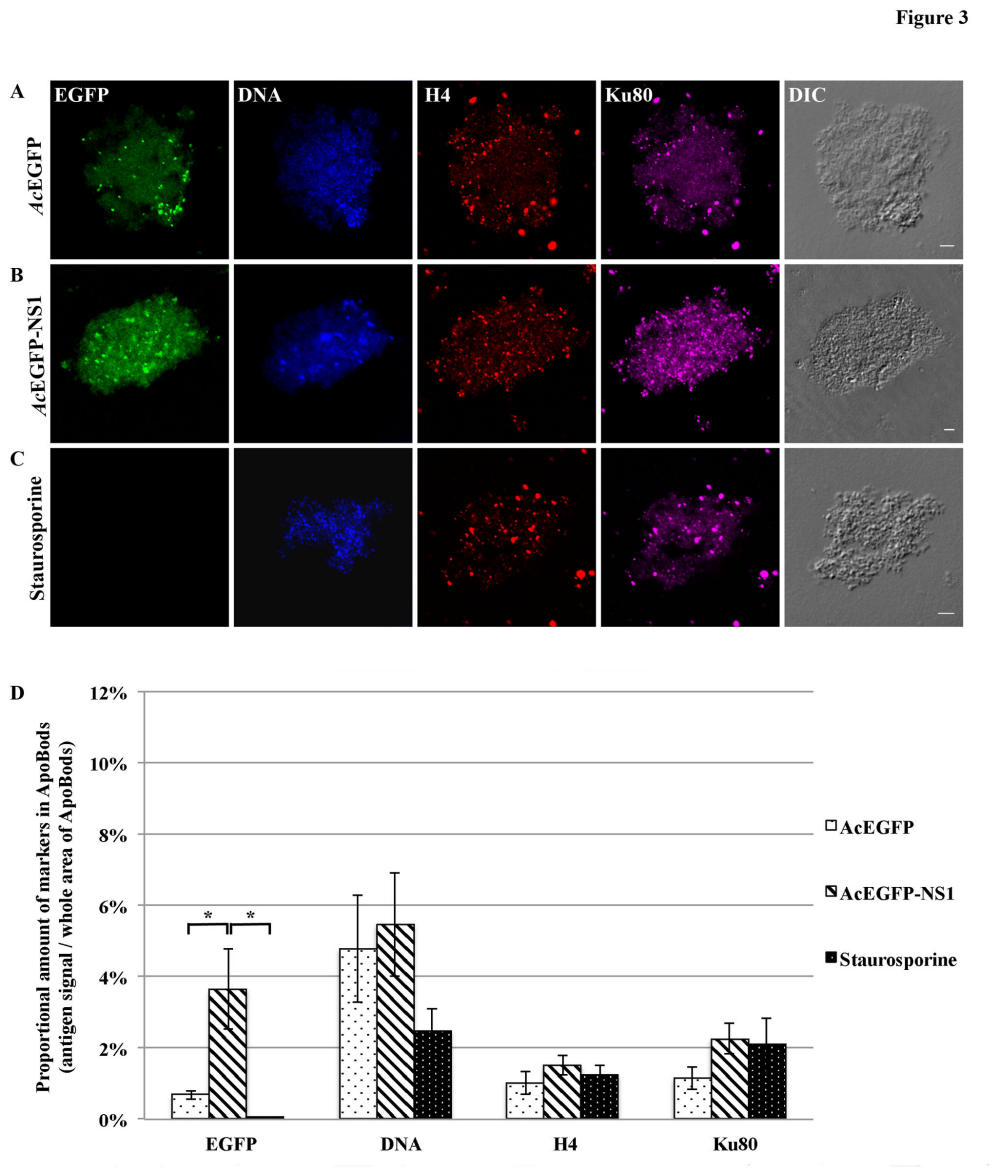
Nuclear antigens histone H4, Ku80, and DNA, are present in NS1 induced ApoBods. Laser scanning confocal images of purified ApoBods produced from with (**A**) *Ac*EGFP, and (**B**) *Ac*EGFP-NS1 transduced cells and (**C**) staurosporine treated HepG2 cells. Purified ApoBods were visualized directly for EGFP (green), stained for DNA with DAPI (blue), immune-labeled for H4 (histone-4; red), and for Ku80 (Ku protein; violet) antigens. DIC microscopy was used to visualize the morphology of the ApoBods. Bars 5 µm. (**D**) amount of antigen markers in ApoBods under different conditions presented as mean ± SEM, N = 30. *P < 0.05.

**Figure 4:**
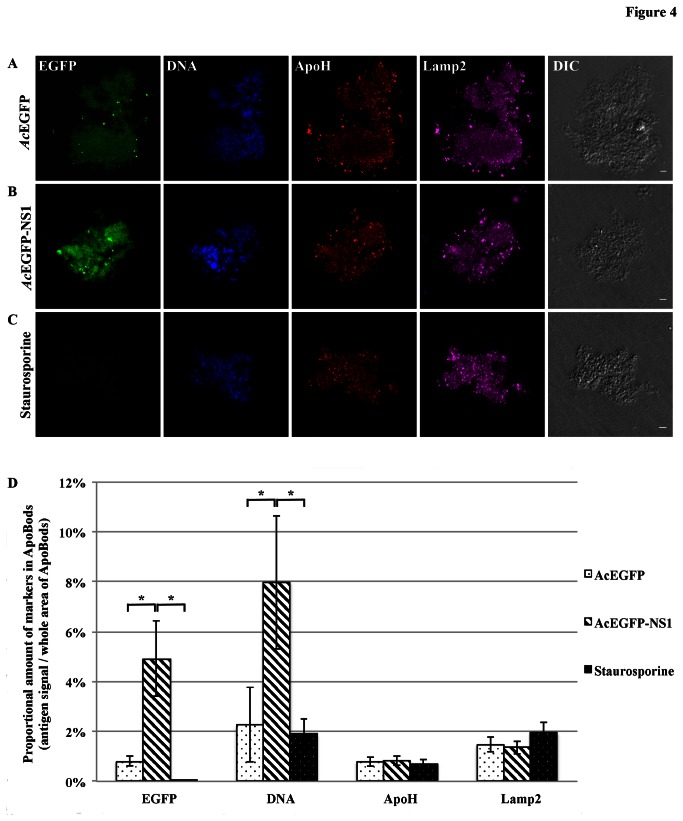
Cytosolic antigens apolipoprotein-H and lysosomal, exist in NS1 induced ApoBods. Confocal microscopy of purified ApoBods produced from with (**A**) *Ac*EGFP, and (**B**) *Ac*EGFP-NS1 transduced cells and (**C**) Staurosporine treated HepG2 cells. Presence of EGFP, DNA, ApoH (apolipoprotein-H), and Lamp2 (lysosomal) antigens are highlighted as green, blue, red and violet, respectively. DIC is displayed to indicate morphology of the ApoBods. Bars 5 µm. The proportion of amount of antigen markers in ApoBods (volume of antigens / total area of ApoBods) was presented as mean ± SEM (N = 30), (**D**). *P < 0.05.

**Figure 5:**
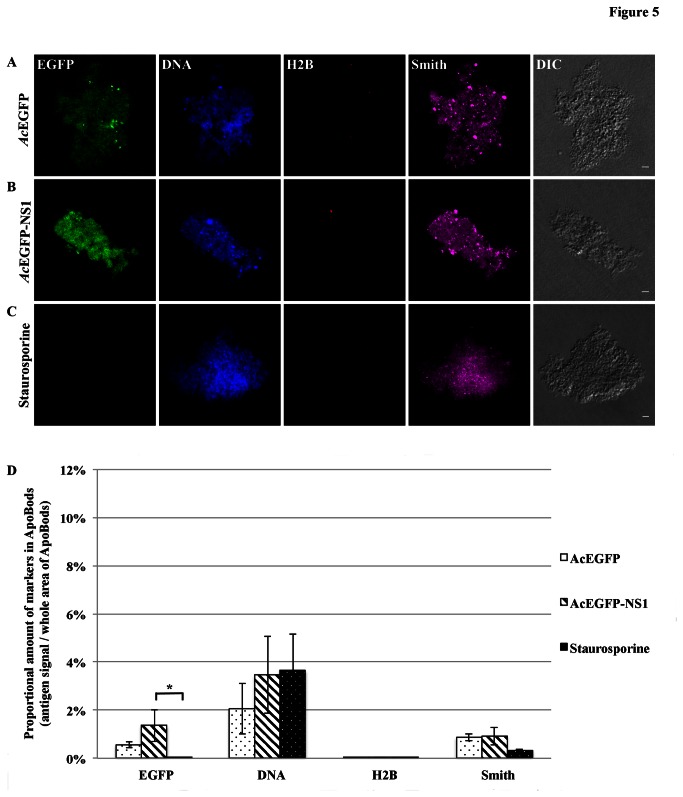
Lupus specific antigens DNA and Smith are seen in purified ApoBods. Confocal microscopy images of purified ApoBods produced from with (**A**) *Ac*EGFP, and (**B**) *Ac*EGFP-NS1 transduced cells and (**C**) Staurosporine treated HepG2 cells. The visualized for EGFP, DNA, H2B and Smith antigens are presented as green, blue, red and violet. Morphology of the ApoBods are seen in the DIC image. Bars 5 µm. Antigen markers of ApoBods from each group was seen in (**D**) by showing as mean ± SEM, N = 30. *P < 0.05.

Additional examinations were conducted with purified ApoBods to identify other self-antigens. Apoptosis was induced in HepG2 cells for 72 h with *Ac*EGFP or *Ac*EGFP-NS1 recombinant baculoviruses, or staurosporine. The purified ApoBods were immunolabeled for nuclear self-antigens histone H4 (seen as red) and Ku80 (viewed as violet) (Figure 3). ApoBods were stained for DNA with Hoechst (blue) and the morphology is seen in the DIC images. Laser scanning confocal microscopy was then used to image the contents of immunolabeled ApoBods and evaluate the volume of antigen signal compared to total area of the DNA signal. Representative confocal images indicated that ApoBods from *Ac*EGFP, *Ac*EGFP-NS1 and staurosporine, Figure 3A–C, respectively, all contain DNA as previously seen. The ApoBods also contained H4 and Ku80, but to a lesser extent in EGFP induced ApoBods. The ApoBods generated by transduction contained nucleosomal antigens as well as NS1. Percentages of antigens in ApoBods are displayed in Figure 3D; *Ac*EGFP-NS1 ApoBods showed highest in all protein signals, including nucleosomal antigens. The EGFP signal was significantly greater than in *Ac*EGFP induced ApoBods (*P* = 0.002) and, as expected, the control staurosporine (*P* = 0.000). Similar labeling experiments were conducted (Figure 4) for apolipoprotein H/beta-2-glycoprotein I (ApoH; red) and lysosomes (Lamp2; violet). These cytosolic constituents were present in the purified ApoBods. Antigens within ApoBods from *Ac*EGFP-NS1 transductions had significantly increased EGFP and DNA signals when compared with *Ac*EGFP ApoBods (*P* = 0.001 for EGFP and *P* = 0.028 for DNA) and staurosporine ApoBods (*P* = 0.000 for EGFP and *P* = 0.033 for DNA), Figure 4D. Purified ApoBods were also labeled for histones (H2B; red) and another nuclear antigen (Smith; violet) (Figure 5). ApoBods from *Ac*EGFP and *Ac*EGFP-NS1 recombinant baculoviruses transduction, and from staurosporine treated cells, did not show the presence of H2B within ApoBods, but Smith was present. In Figure 5D, EGFP signal of *Ac*EGFP-NS1 ApoBods was higher than those from cells transduced with *Ac*EGFP alone (*P* = 0.141) or treated by staurosporine (*P* = 0.014). Moreover, *Ac*EGFP-NS1 ApoBods also presented Smith signal (an SLE marker) even though not at a significantly higher level than *Ac*EGFP. In addition, consistent DNA presence is seen (blue) with these samples, and the characteristic morphology is displayed as viewed in the DIC images. Taken together, specific nucleosomal antigens and specific cytosolic antigens are indeed present in purified ApoBods from NS1 induction.

### Immune cell recognition of purified ApoBods by differentiated macrophages.

**Figure 6:**
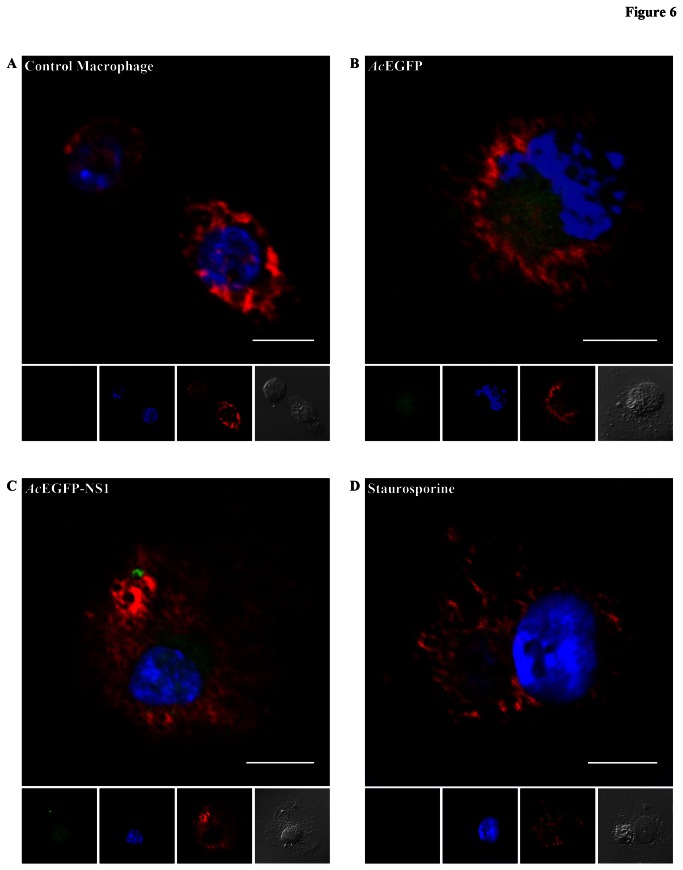
Purified ApoBods induced by NS1 can be engulfed by antigen presenting cells. Laser scanning confocal microscopy images of differentiated macrophages exposed to (**A**) no ApoBods, (**B**) ApoBods from *Ac*EGFP transductions, (**C**) ApoBods from *Ac*EGFP-NS1 transductions, and (**D**) ApoBods produced from staurosporine. Direct viewing from EGFP is seen in green panels, DAPI stained DNA shown as blue, and lysosomes are immunolabeled with Lamp2 antibody with Alexa594 secondary antibody seen as red. DIC frames represent the cell of interest. Merged images represent the compositions of labels seen for the particular treated macrophage. Bars 20 µm.

In order to investigate whether these purified ApoBods would have immunological consequence, differentiated macrophages or differentiated THP-1 cells (dTHP-1) were exposed to the ApoBods from *Ac*EGFP and *Ac*EGFP-NS1 transductions and staurosporine treatment. After 2 h exposure to the ApoBods, the macrophages were washed, fixed and immunolabeled for lysosomes (red) and stained for DNA (blue). Figure 6 represents images from macrophages exposed to no ApoBods (Figure 6A), ApoBods from *Ac*EGFP transductions (Figure 6B), ApoBods produced from transductions of *Ac*EGFP-NS1 (Figure 6C), and ApoBods produced from staurosporine (Figure 6D). The representative confocal images reveal the green fluorescence signal of EGFP (Figure 6B) or EGFP-NS1 (Figure 6C) inside the macrophages. Staurosporine induced ApoBods, as expected (Figure 6D), did not show signal in the green channel. The DIC images depict the morphology of the cell. The larger frame is the merged format of the represented cell. ApoBods from *Ac*EGFP and *Ac*EGFP-NS1 transductions were engulfed, these results were verified by the green signal of ApoBods contained in the macrophages. Thus, engulfment of staurosporine induced ApoBods could not be viewed in this study and hence acted as the negative control for this experiment. In order to further confirm that the ApoBods were internalized, macrophage engulfment inhibitor studies were conducted with the use of cytochalasin B (CB, Figure S1A-B). Confocal images indicated that CB treated dTHP-1 cells engulfed fewer ApoBods from *Ac*EGFP (Figure S1A) and *Ac*EGFP-NS1 (Figure S1B) transductions, as viewed by a lack of green signal inside the cells. When undifferentiated monocytes, THP-1 cells, were exposed to ApoBods from *Ac*EGFP (Figure S1C) and *Ac*EGFP-NS1 (Figure S1D) transductions, or staurosporine treatment (Figure S1E), the cells did not engulf ApoBods as seen by a lack of green signal from the ApoBods in the composition images. The number of cells that engulfed the ApoBods and the phagocytic activities (PA) of differentiated macrophages exposed to the ApoBods were calculated and presented in Table 1. From 1200 cells, 28.6% of the macrophages recognized and engulfed the ApoBods from *Ac*EGFP transductions; this was similar to *Ac*EGFP-NS1 transductions at 26.3% (*P* = 0.219). Uptake was inhibited approximately 56.0% when cytochalasin B was present. Similarly, the macrophages engulfed ApoBods from *Ac*EGFP-NS1 inductions was inhibited by 59.0% in the presence of CB. As expected ApoBods from the staurosporine treatment provided the null values in this experiment.

**Table 1:** Phagocytoic activities of differentiated monocytes exposed to ApoBods.

**Apoptotic bodies(ApoBods)**	**No. of macrophages that engulfed ApoBods(200 cells x 6)**	**PA (%)**	**No. of macrophages that engulfed ApoBods with cytochalasin B(200 cells x 6)**	**PA (%)**	**Inhibited % with cytochalasin B**
**Without**	0	0	Not Applicable	-	-
***Ac*EGFP transduction**	57.2 ± 3.7	28.6	25.2 ± 2.3	12.6	56
***Ac*EGFP-NS1 transduction**	52.5 ± 2.4	26.3	21.8 ± 1.9	10.9	59
**Staurosporine**	0	0	0	0	0

Macrophages exposed to ApoBods from different treatments, without ApoBods, from *Ac*EGFP transductions, transductions of *Ac*EGFP-NS1, and produced from staurosporine, were evaluated regarding the number of cells that engulfed the ApoBods. The phagocytic activity was calculated according to the following formula: PA% = (number of macrophages containing engulfed ApoBods/total number of counted macrophages) × 100. Phagocytotic cells with the preceding ApoBods were treated with CB. The number of cells that engulfed the ApoBods and the number of inhibition percentage are given. Engulfed ApoBods were counted for 200 cells x 6, total N = 1,200 cells.

## Discussion

### NS1 protein stimulate ApoBods production

The mechanisms by which viruses break immune tolerance require further investigation. Apoptosis is an intracellular death pathway to remove cells without provoking a response to cell debris [52,53]. Morphological evidence of apoptosis consists of cell shrinkage, chromatin condensation, membrane blebbing, and the formation of ApoBods [53,54] as seen also here (Figure 1). The important roles of apoptosis include the regulation of hematopoetic progenitor cells, the elimination of cells that have sustained genetic damage or that undergo uncontrolled cellular proliferation, and the prevention of viral replication [55]. Characteristically, the loss of phosphatidylserine (PS) from the intercellular surface to the outer layer of the plasma lipid bilayer membrane occurs in the early stages of apoptosis [56,57]. During advanced stages of apoptosis, intracellular fragments independently move to the cell surface creating two discrete structures, namely surface blebs then ApoBods that actually separate from the remainder cell [57]. Our results demonstrated that B19V NS1 protein in a non-permissive cell line induces high quantities of apoptotic blebs (Figure 1) and bodies that contain NS1 protein and exposed PS (Figure 2). These NS1 protein induced apoptotic events have been reported earlier [15].

### Detection of self-antigens and viral proteins

Apoptotic bleb/bodies usually contain only self-antigens; these self-antigens typically do not cause inflammatory or immune responses [52,53,56]. Impaired clearance of apoptotic cells has been proposed to cause autoimmunity by increasing the quantity of ApoBods and expanding the diversity of self-antigens presented to the immune system [58]. In systemic immune diseases such like SLE, antibodies to self-antigens may be used for diagnosis including circulating antibodies to DNA, nuclear fragments and histones [58,59]. B19V NS1 has been reported to provoke cellular DNA damage [13,18,49,60]. Other studies have detected autoantibodies to self-antigens post B19V infection [46,61]. Several studies have hypothesized that the mechanism of B19V induced autoimmunity is due to molecular mimicry [62–64]. Here, we confirm that the purified ApoBods have self-antigens from the cytoplasm and nucleus (Figures 2-5). In addition, some specific autoimmune disease biomarker autoantibody targets such as DNA, H4, Ku80, ApoH and Smith are present (Figures 3-5). While it has previously been shown that high amounts of EGFP alone expressed in recombinant systems can adversely affect cell physiology [65], which can lead to apoptosis [66], and components of ApoBods from *Ac*EGFP and *Ac*EGFP-NS1 transductions were qualitatively quit similar, quantitatively there were 2-fold fewer ApoBods produced from *Ac*EGFP transduction.

Established clinical viral infections, as in the case with herpes simplex virus 1 (HSV-1) [67], hepatitis C virus (HCV) [68], Epstein–Barr virus (EBV) [69], and cytomegalovirus (CMV) [70], have been reported to induce or exacerbate autoimmune diseases. At least three mechanisms have been proposed to explain this exacerbation: (1) the immune response to pathogens provides a specific or nonspecific stimulus that promotes activation and expansion of auto-reactive T cells, (2) the viral pathogen itself may provide a potential role of antigenic stimulus to provoke auto-reactive T cells, and (3) molecular mimicry of a viral epitope may allow self-antigen expression that can be taken up, processed and cross-presented by APCs [67,71,72].

### Engulfment of ApoBods

Generally, apoptotic cells are cleared rapidly and efficiently as intact cells or ApoBods by professional antigen presenting cells (APCs) or neighboring cells [52–54,56]. This engulfment typically does not elicit an inflammatory or immune responses [53]. Self-antigen translocation can enhance phagocytosis of apoptotic fragments and presentation of autoantigens [73,74]. We have shown here that the ApoBods are recognized and engulfed by differentiated macrophages (Figure 6 Table 1 Figure S1F), and this suggests that the B19V induced ApoBods have the potential to provide a repertoire of self-antigens to the immune system. It has been previously reported that deficiencies of apoptotic clearance processes may expose the immune system to more advanced stages of apoptotic structures [58,74,75]. In apoptosis, intracellular fragments independently move to the cell surface creating two discrete structures, apoptotic blebs then bodies that actually separate from the remainder cell [57]. These structures serve as autoantigens generating autoantibodies [76]. Nucleosomal NS1 protein modified DNA may contain additional nuclear antigens as DNA binding protein or NS1 interactive proteins. Uptake of this complex by anergic B lymphocytes specific for DNA or self-antigen would allow presentation of NS1 peptides to NS1 specific T helper cells, thereby breaking tolerance. Reaction to self- and nonself-antigens by APCs and lymphocytes would elicit tissue damage that in turn accelerates autoimmune disease [77]. Therefore, immune processing of B19V NS1 protein induced ApoBods warrants further investigation.

## Supporting Information

Figure S1 Laser scanning confocal microscopy images of the engulfment study that showed no phagocytosis. Macrophages with the inhibitor cytochalasin B (CB) were exposed to ApoBods from the following productions (**A**) *Ac*EGFP and (**B**) *Ac*EGFP-NS1 transductions. Monocytes exposed to ApoBods from (**C**) *Ac*EGFP transductions, (**D**) *Ac*EGFP-NS1 transductions, and (**E**) staurosporine treatment. (**F**) positive control is macrophages exposed to ApoBods from *Ac*EGFP-NS1 transduction. EGFP signal is viewed directly in the green frame, DNA is stain with DAPI seen in the blue images, and lysosomes depicted as red when labeled with Lamp2 antibody. Compositions of the labels are seen in merged images. DIC represents the morphology of the cells. Bars 20 µm.

Table S1 Purified ApoBods in consequence of NS1 expression presented high quantity and green signal. Quantity of purified ApoBods from transduced cells with *Ac*EGFP and *Ac*EGFP-NS1, and treated with staurosporine control from FC 3 different assays were analyzed. The results from each condition presented as mean ± SEM (N = 3). *P* value < 0.05 is significantly; *compare between *Ac*EGFP and *Ac*EGFP-NS1, **compare between *Ac*EGFP and staurosporine, and ***compare between *Ac*EGFP-NS1 and staurosporine.

## References

[1] Clewley JP (1984) Biochemical characterization of a human parvovirus. J Gen Virol 65(1): 241-245. doi:10.1099/0022-1317-65-1-241. 6693855 10.1099/0022-1317-65-1-241

[2] Cossart YE, Field AM, Cant B, Widdows D (1975) Parvovirus-like particles in human sera. Lancet 1: 72-73. PubMed: 46024. 46024 10.1016/s0140-6736(75)91074-0

[3] Shade RO, Blundell MC, Cotmore SF, Tattersall P, Astell CR (1986) Nucleotide sequence and genome organization of human parvovirus B19 isolated from the serum of a child during aplastic crisis. J Virol 58: 921-936. PubMed: 3701931. 3701931 10.1128/jvi.58.3.921-936.1986 PMC253001

[4] Young NS, Brown KE (2004) Parvovirus B19. N Engl J Med 350: 586-597. doi:10.1056/NEJMra030840. PubMed: 14762186. 14762186 10.1056/NEJMra030840

[5] Zádori Z, Szelei J, Lacoste MC, Li Y, Gariépy S et al. (2001) A viral phospholipase A2 is required for parvovirus infectivity. Dev Cell 1: 291-302. doi:10.1016/S1534-5807(01)00031-4. PubMed: 11702787. 11702787 10.1016/s1534-5807(01)00031-4

[6] Raab U, Beckenlehner K, Lowin T, Niller HH, Doyle S et al. (2002) NS1 protein of parvovirus B19 interacts directly with DNA sequences of the p6 promoter and with the cellular transcription factors Sp1/Sp3. Virology 293: 86-93. doi:10.1006/viro.2001.1285. PubMed: 11853402. 11853402 10.1006/viro.2001.1285

[7] Fu Y, Ishii KK, Munakata Y, Saitoh T, Kaku M et al. (2002) Regulation of tumor necrosis factor alpha promoter by human parvovirus B19 NS1 through activation of AP-1 and AP-2. J Virol 76: 5395-5403. doi:10.1128/JVI.76.11.5395-5403.2002. PubMed: 11991968. 11991968 10.1128/JVI.76.11.5395-5403.2002 PMC137035

[8] Mitchell LA (2002) Parvovirus B19 nonstructural (NS1) protein as a transactivator of interleukin-6 synthesis: common pathway in inflammatory sequelae of human parvovirus infections? J Med Virol 67: 267-274. doi:10.1002/jmv.2217. PubMed: 11992589. 11992589 10.1002/jmv.2217

[9] Young N, Harrison M, Moore J, Mortimer P, Humphries RK (1984) Direct demonstration of the human parvovirus in erythroid progenitor cells infected in vitro. J Clin Invest 74: 2024-2032. doi:10.1172/JCI111625. PubMed: 6392340. 6392340 10.1172/JCI111625 PMC425391

[10] Ozawa K, Kurtzman G, Young N (1987) Productive infection by B19 parvovirus of human erythroid bone marrow cells in vitro. Blood 70: 384-391. PubMed: 3038211. 3038211

[11] Srivastava A, Bruno E, Briddell R, Cooper R, Srivastava C et al. (1990) Parvovirus B19-induced perturbation of human megakaryocytopoiesis in vitro. Blood 76: 1997-2004. PubMed: 2146978. 2146978

[12] Munshi NC, Zhou S, Woody MJ, Morgan DA, Srivastava A (1993) Successful replication of parvovirus B19 in the human megakaryocytic leukemia cell line MB-02. J Virol 67: 562-566. PubMed: 8416383. 8416383 10.1128/jvi.67.1.562-566.1993 PMC237395

[13] Moffatt S, Yaegashi N, Tada K, Tanaka N, Sugamura K (1998) Human parvovirus B19 nonstructural (NS1) protein induces apoptosis in erythroid lineage cells. J Virol 72: 3018-3028. PubMed: 9525624. 9525624 10.1128/jvi.72.4.3018-3028.1998 PMC109749

[14] Sol N, Le Junter J, Vassias I, Freyssinier JM, Thomas A et al. (1999) Possible interactions between the NS-1 protein and tumor necrosis factor alpha pathways in erythroid cell apoptosis induced by human parvovirus B19. J Virol 73: 8762-8770. PubMed: 10482630. 10482630 10.1128/jvi.73.10.8762-8770.1999 PMC112897

[15] Poole BD, Karetnyi YV, Naides SJ (2004) Parvovirus B19-induced apoptosis of hepatocytes. J Virol 78: 7775-7783. doi:10.1128/JVI.78.14.7775-7783.2004. PubMed: 15220451. 15220451 10.1128/JVI.78.14.7775-7783.2004 PMC434113

[16] Poole BD, Zhou J, Grote A, Schiffenbauer A, Naides SJ (2006) Apoptosis of liver-derived cells induced by parvovirus B19 nonstructural protein. J Virol 80: 4114-4121. doi:10.1128/JVI.80.8.4114-4121.2006. PubMed: 16571827. 16571827 10.1128/JVI.80.8.4114-4121.2006 PMC1440431

[17] Kivovich V, Gilbert L, Vuento M, Naides SJ (2010) Parvovirus B19 genotype specific amino acid substitution in NS1 reduces the protein’s cytotoxicity in culture. Int J Med Sci 7: 110-119. PubMed: 20567611. 20567611 10.7150/ijms.7.110 PMC2880839

[18] Kivovich V, Gilbert L, Vuento M, Naides SJ (2012) The putative metal coordination motif in the endonuclease domain of human Parvovirus B19 NS1 is critical for NS1 induced S phase arrest and DNA damage. Int J Biol Sci 8: 79-92. doi:10.3923/ijb.2012.79.81. PubMed: 22211107. 22211107 10.7150/ijbs.8.79 PMC3248650

[19] Bonvicini F, Filippone C, Manaresi E, Zerbini M, Musiani M et al. (2008) HepG2 hepatocellular carcinoma cells are a non-permissive system for B19 virus infection. J Gen Virol 89: 3034-3038. doi:10.1099/vir.0.2008/004341-0. PubMed: 19008390. 19008390 10.1099/vir.0.2008/004341-0

[20] Kerr JR (1996) Parvovirus B19 infection. Eur J Clin Microbiol Infect Dis 15: 10-29. doi:10.1007/BF01586181. PubMed: 8641299. 8641299 10.1007/BF01586181

[21] Kerr S, O’Keeffe G, Kilty C, Doyle S (1999) Undenatured parvovirus B19 antigens are essential for the accurate detection of parvovirus B19 IgG. J Med Virol 57: 179-185. doi:10.1002/(SICI)1096-9071(199902)57:2. PubMed: 9892405. 9892405 10.1002/(sici)1096-9071(199902)57:2<179::aid-jmv16>3.0.co;2-t

[22] Anderson MJ, Cohen BJ (1987) Human Parvovirus B19 Infections in United-Kingdom 1984-86. Lancet 1: 738-739. 2882143

[23] Heegaard ED, Brown KE (2002) Human parvovirus B19. Clin Microbiol Rev 15: 485-505. doi:10.1128/CMR.15.3.485-505.2002. PubMed: 12097253. 12097253 10.1128/CMR.15.3.485-505.2002 PMC118081

[B24] Anderson MJ, Higgins PG, Davis LR, Willman JS, Jones SE et al. (1985) Experimental Parvoviral Infection in Humans. J Infect Dis 152: 257-265. doi:10.1093/infdis/152.2.257. PubMed: 2993431. 2993431 10.1093/infdis/152.2.257

[25] Potter CG, Potter AC, Hatton CS, Chapel HM, Anderson MJ et al. (1987) Variation of erythroid and myeloid precursors in the marrow and peripheral blood of volunteer subjects infected with human parvovirus (B19). J Clin Invest 79: 1486-1492. doi:10.1172/JCI112978. PubMed: 3033026. 3033026 10.1172/JCI112978 PMC424424

[26] Lehmann HW, von Landenberg P, Modrow S (2003) Parvovirus B19 infection and autoimmune disease. Autoimmun Rev 2: 218-223. doi:10.1016/S1568-9972(03)00014-4. PubMed: 12848949. 12848949 10.1016/s1568-9972(03)00014-4

[27] Corcoran A, Doyle S (2004) Advances in the biology, diagnosis and host-pathogen interactions of parvovirus B19. J Med Microbiol 53: 459-475. doi:10.1099/jmm.0.05485-0. PubMed: 15150324. 15150324 10.1099/jmm.0.05485-0

[28] Drago F, Semino M, Rampini P, Rebora A (1999) Parvovirus B19 infection associated with acute hepatitis and a purpuric exanthem. Br J Dermatol 141: 160-161. doi:10.1046/j.1365-2133.1999.02943.x. PubMed: 10417538. 10417538 10.1046/j.1365-2133.1999.02943.x

[29] Yang SH, Lin LW, Fang YJ, Cheng AL, Kuo SH (2012) Parvovirus B19 infection-related acute hepatitis after rituximab-containing regimen for treatment of diffuse large B-cell lymphoma. Ann Hematol 91: 291-294. doi:10.1007/s00277-011-1238-8. PubMed: 21538062. 21538062 10.1007/s00277-011-1238-8

[30] Pinho JR, Alves VA, Vieira AF, Moralez MO, Fonseca LE et al. (2001) Detection of human parvovirus B19 in a patient with hepatitis. Braz J Med Biol Res 34: 1131-1138. PubMed: 11514836. 11514836 10.1590/s0100-879x2001000900005

[31] Arista S, De Grazia S, Di Marco V, Di Stefano R, Craxì A (2003) Parvovirus B19 and "cryptogenic" chronic hepatitis. J Hepatol 38: 375-376. doi:10.1016/S0270-9139(03)80488-3. PubMed: 12586308. 12586308 10.1016/s0168-8278(02)00416-6

[32] Mogensen TH, Jensen JM, Hamilton-Dutoit S, Larsen CS (2010) Chronic hepatitis caused by persistent parvovirus B19 infection. BMC Infect Dis 10: 246. doi:10.1186/1471-2334-10-246. PubMed: 20727151. 20727151 10.1186/1471-2334-10-246 PMC2936411

[33] Nobili V, Vento S, Comparcola D, Sartorelli MR, Luciani M et al. (2004) Autoimmune hemolytic anemia and autoimmune hepatitis associated with parvovirus B19 infection. Pediatr Infect Dis J 23: 184-185. doi:10.1097/01.inf.0000110270.38240.51. PubMed: 14872194. 14872194 10.1097/01.inf.0000110270.38240.51

[34] Kordes U, Schneppenheim R, Briem-Richter A, Scherpe S, Schäfer HJ (2011) Parvovirus B19 infection and autoimmune hepatitis in a child with sickle cell anemia. Pediatr Blood Cancer 56: 323-324. doi:10.1002/pbc.22820. PubMed: 21157899. 21157899 10.1002/pbc.22820

[35] Díaz F, Collazos J (2000) Hepatic dysfunction due to parvovirus B19 infection. J Infect Chemother 6: 63-64. doi:10.1007/s101560050052. PubMed: 11810534. 11810534 10.1007/s101560050052

[36] Dame C, Hasan C, Bode U, Eis-Hübinger AM (2002) Acute liver disease and aplastic anemia associated with the persistence of B19 DNA in liver and bone marrow. Pediatr Pathol Mol Med 21: 25-29. doi:10.1080/pdp.21.1.25.29. PubMed: 11842976. 11842976 10.1080/pdp.21.1.25.29

[37] Krygier DS, Steinbrecher UP, Petric M, Erb SR, Chung SW et al. (2009) Parvovirus B19 induced hepatic failure in an adult requiring liver transplantation. World J Gastroenterol 15: 4067-4069. doi:10.3748/wjg.15.4067. PubMed: 19705505. 19705505 10.3748/wjg.15.4067 PMC2731960

[38] Karetnyi YV, Beck PR, Markin RS, Langnas AN, Naides SJ (1999) Human parvovirus B19 infection in acute fulminant liver failure. Arch Virol 144: 1713-1724. doi:10.1007/s007050050699. PubMed: 10542021. 10542021 10.1007/s007050050699

[39] Abe K, Kiuchi T, Tanaka K, Edamoto Y, Aiba N et al. (2007) Characterization of erythrovirus B19 genomes isolated in liver tissues from patients with fulminant hepatitis and biliary atresia who underwent liver transplantation. Int J Med Sci 4: 105-109. PubMed: 17479159. 17479159 10.7150/ijms.4.105 PMC1852398

[40] Sun L, Zhang JC (2012) Acute fulminant hepatitis with bone marrow failure in an adult due to parvovirus B19 infection. Hepatology 55: 329-330. doi:10.1002/hep.24720. PubMed: 21969057. 21969057 10.1002/hep.24720

[41] Hemauer A, Beckenlehner K, Wolf H, Lang B, Modrow S (1999) Acute parvovirus B19 infection in connection with a flare of systemic lupus erythematodes in a female patient. J Clin Virol 14: 73-77. doi:10.1016/S1386-6532(99)00038-4. PubMed: 10548133. 10548133 10.1016/s1386-6532(99)00038-4

[42] Takahashi Y, Murai C, Shibata S, Munakata Y, Ishii T et al. (1998) Human parvovirus B19 as a causative agent for rheumatoid arthritis. Proc Natl Acad Sci U S A 95: 8227-8232. doi:10.1073/pnas.95.14.8227. PubMed: 9653169. 9653169 10.1073/pnas.95.14.8227 PMC20958

[43] Dorsch S, Liebisch G, Kaufmann B, von Landenberg P, Hoffmann JH et al. (2002) The VP1 unique region of parvovirus B19 and its constituent phospholipase A2-like activity. J Virol 76: 2014-2018. doi:10.1128/JVI.76.4.2014-2018.2002. PubMed: 11799199. 11799199 10.1128/JVI.76.4.2014-2018.2002 PMC135890

[44] von Landenberg P, Lehmann HW, Knöll A, Dorsch S, Modrow S (2003) Antiphospholipid antibodies in pediatric and adult patients with rheumatic disease are associated with parvovirus B19 infection. Arthritis Rheum 48: 1939-1947. doi:10.1002/art.11038. PubMed: 12847688. 12847688 10.1002/art.11038

[45] von Landenberg P, Lehmann HW, Modrow S (2007) Human parvovirus B19 infection and antiphospholipid antibodies. Autoimmun Rev 6: 278-285. doi:10.1016/j.autrev.2006.09.006. PubMed: 17412298. 17412298 10.1016/j.autrev.2006.09.006

[46] Lunardi C, Tinazzi E, Bason C, Dolcino M, Corrocher R et al. (2008) Human parvovirus B19 infection and autoimmunity. Autoimmun Rev 8: 116-120. doi:10.1016/j.autrev.2008.07.005. PubMed: 18700174. 18700174 10.1016/j.autrev.2008.07.005

[47] Lunardi C, Tiso M, Borgato L, Nanni L, Millo R et al. (1998) Chronic parvovirus B19 infection induces the production of anti-virus antibodies with autoantigen binding properties. Eur J Immunol 28: 936-948. doi:10.1002/(SICI)1521-4141(199803)28:03. PubMed: 9541589. 9541589 10.1002/(SICI)1521-4141(199803)28:03<936::AID-IMMU936>3.0.CO;2-X

[48] Moffatt S, Tanaka N, Tada K, Nose M, Nakamura M et al. (1996) A cytotoxic nonstructural protein, NS1, of human parvovirus B19 induces activation of interleukin-6 gene expression. J Virol 70: 8485-8491. PubMed: 8970971. 8970971 10.1128/jvi.70.12.8485-8491.1996 PMC190939

[49] Poole BD, Kivovich V, Gilbert L, Naides SJ (2011) Parvovirus B19 nonstructural protein-induced damage of cellular DNA and resultant apoptosis. Int J Med Sci 8: 88-96. PubMed: 21278893. 21278893 10.7150/ijms.8.88 PMC3030141

[50] Daigneault M, Preston JA, Marriott HM, Whyte MK, Dockrell DH (2010) The identification of markers of macrophage differentiation in PMA-stimulated THP-1 cells and monocyte-derived macrophages. PLOS ONE 5: e8668. doi:10.1371/journal.pone.0008668. PubMed: 20084270. 20084270 10.1371/journal.pone.0008668 PMC2800192

[51] Kankaanpää P, Paavolainen L, Tiitta S, Karjalainen M, Päivärinne J et al. (2012) BioImageXD: An open, general-purpose and high-throughput image-processing platform. Nat Methods 9: 683-689. doi:10.1038/nmeth.2047. PubMed: 22743773. 22743773 10.1038/nmeth.2047

[52] Kerr JF, Wyllie AH, Currie AR (1972) Apoptosis: a basic biological phenomenon with wide-ranging implications in tissue kinetics. Br J Cancer 26: 239-257. doi:10.1038/bjc.1972.33. PubMed: 4561027. 4561027 10.1038/bjc.1972.33 PMC2008650

[53] Arends MJ, Wyllie AH (1991) Apoptosis: mechanisms and roles in pathology. Int Rev Exp Pathol 32: 223-254. PubMed: 1677933. 1677933 10.1016/b978-0-12-364932-4.50010-1

[54] Krysko DV, Denecker G, Festjens N, Gabriels S, Parthoens E et al. (2006) Macrophages use different internalization mechanisms to clear apoptotic and necrotic cells. Cell Death Differ 13: 2011-2022. doi:10.1038/sj.cdd.4401900. PubMed: 16628234. 16628234 10.1038/sj.cdd.4401900

[55] Barber GN (2001) Host defense, viruses and apoptosis. Cell Death Differ 8: 113-126. doi:10.1038/sj.cdd.4400823. PubMed: 11313713. 11313713 10.1038/sj.cdd.4400823

[56] Radic M, Marion T, Monestier M (2004) Nucleosomes are exposed at the cell surface in apoptosis. J Immunol 172: 6692-6700. PubMed: 15153485. 15153485 10.4049/jimmunol.172.11.6692

[57] Casciola-Rosen LA, Anhalt G, Rosen A (1994) Autoantigens targeted in systemic lupus erythematosus are clustered in two populations of surface structures on apoptotic keratinocytes. J Exp Med 179: 1317-1330. doi:10.1084/jem.179.4.1317. PubMed: 7511686. 7511686 10.1084/jem.179.4.1317 PMC2191465

[58] Cline AM, Radic MZ (2004) Apoptosis, subcellular particles, and autoimmunity. Clin Immunol 112: 175-182. doi:10.1016/j.clim.2004.02.017. PubMed: 15240161. 15240161 10.1016/j.clim.2004.02.017

[59] Fadok VA, Voelker DR, Campbell PA, Cohen JJ, Bratton DL et al. (1992) Exposure of Phosphatidylserine on the Surface of Apoptotic Lymphocytes Triggers Specific Recognition and Removal by Macrophages. J Immunol 148: 2207-2216. PubMed: 1545126. 1545126

[60] Ozawa K, Ayub J, Kajigaya S, Shimada T, Young N (1988) The Gene Encoding the Nonstructural Protein of B19 (Human) Parvovirus May Be Lethal in Transfected Cells. J Virol 62: 2884-2889. PubMed: 2969055. 2969055 10.1128/jvi.62.8.2884-2889.1988 PMC253725

[61] Meyer O (2003) Parvovirus B19 and autoimmune diseases. Joint Bone Spine 70: 6-11. doi:10.1016/S1297-319X(02)00004-0. PubMed: 12639611. 12639611 10.1016/s1297-319x(02)00004-0

[62] Murphy PM (2001) Viral exploitation and subversion of the immune system through chemokine mimicry. Nat Immunol 2: 116-122. doi:10.1038/84214. PubMed: 11175803. 11175803 10.1038/84214

[B63] Alcami A (2003) Viral mimicry of cytokines, chemokines and their receptors. Nat Rev Immunol 3: 36-50. doi:10.1038/nri980. PubMed: 12511874. 12511874 10.1038/nri980

[64] Bruggeman LA (2007) Viral subversion mechanisms in chronic kidney disease pathogenesis. Clin J Am Soc Nephrol 2 Suppl 1: S13-S19. doi:10.2215/CJN.04311206. PubMed : 17699505 10.2215/CJN.04311206 PMC2212606 17699505

[65] Baens M, Noels H, Broeckx V, Hagens S, Fevery S et al. (2006) The Dark Side of EGFP: Defective Polyubiquitination. PLOS ONE 1: e54. doi:10.1371/journal.pone.0000054. PubMed: 17183684. 17183684 10.1371/journal.pone.0000054 PMC1762387

[66] Liu HS, Jan MS, Chou CK, Chen PH, Ke NJ (1999) Is green fluorescent protein toxic to the living cells? Biochem Biophys Res Commun 260: 712-717. doi:10.1006/bbrc.1999.0954. PubMed: 10403831. 10403831 10.1006/bbrc.1999.0954

[67] Panoutsakopoulou V, Sanchirico ME, Huster KM, Jansson M, Granucci F et al. (2001) Analysis of the relationship between viral infection and autoimmune disease. Immunity 15: 137-147. doi:10.1016/S1074-7613(01)00172-8. PubMed: 11485745. 11485745 10.1016/s1074-7613(01)00172-8

[68] Wagner B, Vierhapper H, Hofmann H (1996) Prevalence of hepatitis C virus infection in Hashimoto’s thyroiditis. BMJ 312: 640-641. doi:10.1136/bmj.312.7031.640b. PubMed: 8595364. 10.1136/bmj.312.7031.640b PMC2350409 8595364

[69] Vento S, Guella L, Mirandola F, Cainelli F, Di Perri G et al. (1995) Epstein-Barr virus as a trigger for autoimmune hepatitis in susceptible individuals. Lancet 346: 608-609. doi:10.1016/S0140-6736(95)91438-2. PubMed: 7651006. 7651006 10.1016/s0140-6736(95)91438-2

[70] Lawson CM, O’Donoghue HL, Reed WD (1992) Mouse cytomegalovirus infection induces antibodies which cross-react with virus and cardiac myosin: A model for the study of molecular mimicry in the pathogenesis of viral myocarditis. Immunology 75: 513-519. PubMed: 1315309. 1315309 PMC1384748

[71] von Herrath MG, Oldstone MB (1996) Virus-induced autoimmune disease. Curr Opin Immunol 8: 878-885. doi:10.1016/S0952-7915(96)80019-7. PubMed: 8994870. 8994870 10.1016/S0952-7915(96)80019-7 PMC7134972

[72] Wraith DC, Goldman M, Lambert PH (2003) Vaccination and autoimmune disease: what is the evidence? Lancet 362: 1659-1666. doi:10.1016/S0140-6736(03)14802-7. PubMed: 14630450. 14630450 10.1016/S0140-6736(03)14802-7

[73] Frisoni L, McPhie L, Colonna L, Sriram U, Monestier M et al. (2005) Nuclear autoantigen translocation and autoantibody opsonization lead to increased dendritic cell phagocytosis and presentation of nuclear antigens: a novel pathogenic pathway for autoimmunity? J Immunol 175: 2692-2701. PubMed: 16081846. 16081846 10.4049/jimmunol.175.4.2692

[74] Schiller M, Bekeredjian-Ding I, Heyder P, Blank N, Ho AD et al. (2008) Autoantigens are translocated into small apoptotic bodies during early stages of apoptosis. Cell Death Differ 15: 183-191. doi:10.1038/sj.cdd.4402239. PubMed: 17932498. 17932498 10.1038/sj.cdd.4402239

[75] Cline AM, Radic MZ (2004) Murine lupus autoantibodies identify distinct subsets of apoptotic bodies. Autoimmunity 37: 85-93. doi:10.1080/0891693042000196219. PubMed: 15293878. 15293878 10.1080/0891693042000196219

[76] Cocca BA, Cline AM, Radic MZ (2002) Blebs and apoptotic bodies are B cell autoantigens. J Immunol 169: 159-166. PubMed: 12077241. 12077241 10.4049/jimmunol.169.1.159

[77] Murphy K, Travers P, Walport M (2008) Janeway’s immunobiology. New York Garlan. Science Publishing House.

