# Supplementary figures and images for: Human Parvovirus B19 Induced Apoptotic Bodies Contain Altered Self-Antigens that are Phagocytosed by Antigen Presenting Cells
Source: PLoS One. 2013 Jun 12;8(6):e67179. doi: 10.1371/journal.pone.0067179 (PMC3680405; doi:10.1371/journal.pone.0067179)

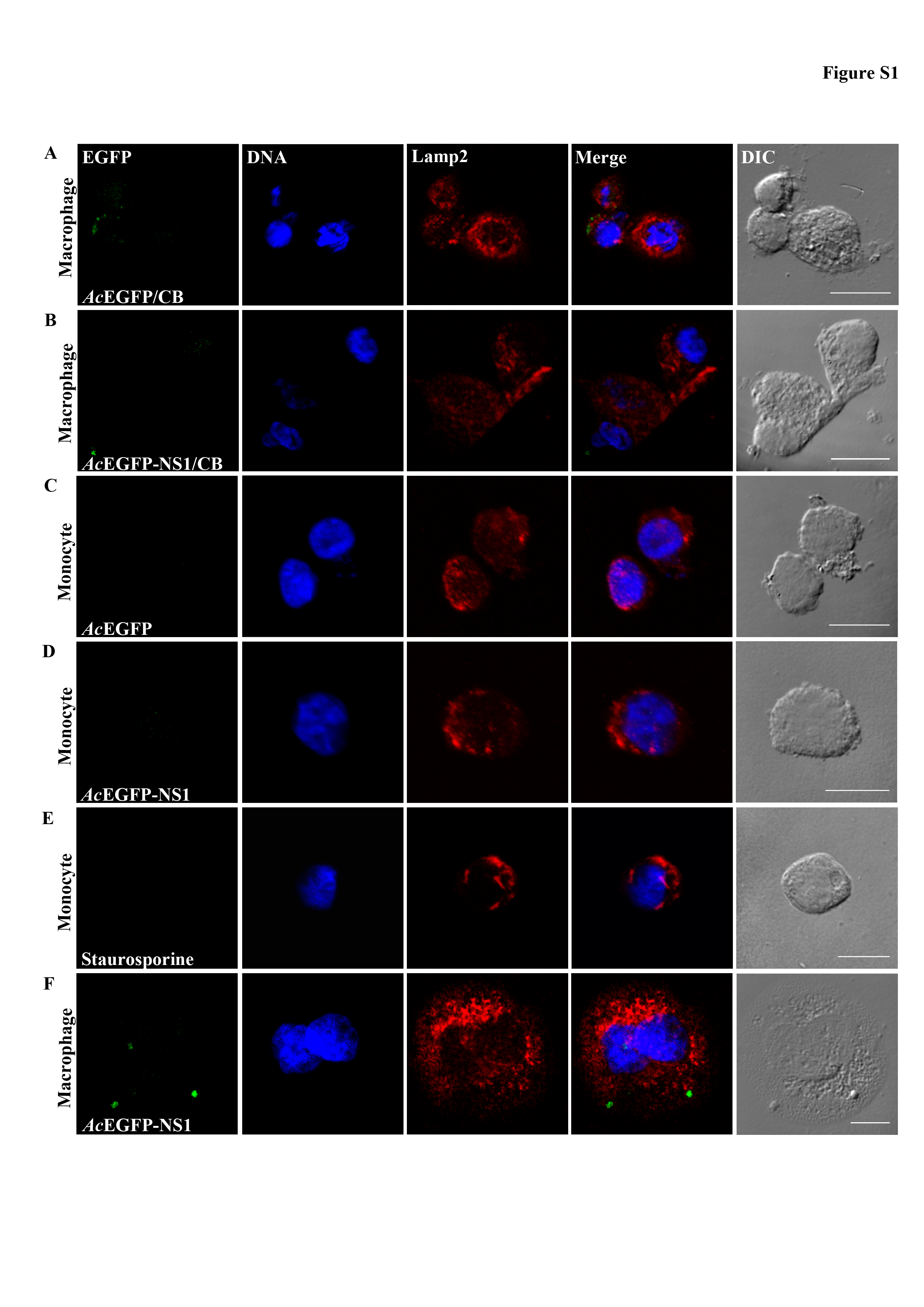

Supplement: Figure S1 — Laser scanning confocal microscopy images of the engulfment study that showed no phagocytosis. Macrophages with the inhibitor cytochalasin B (CB) were exposed to ApoBods from the following productions (A) AcEGFP and (B) AcEGFP-NS1 transductions. Monocytes exposed to ApoBods from (C) AcEGFP transductions, (D) AcEGFP-NS1 transductions, and (E) staurosporine treatment. (F) positive control is macrophages exposed to ApoBods from AcEGFP-NS1 transduction. EGFP signal is viewed directly in the green frame, DNA is stain with DAPI seen in the blue images, and lysosomes depicted as red when labeled with Lamp2 antibody. Compositions of the labels are seen in merged images. DIC represents the morphology of the cells. Bars 20 µm. [file pone.0067179.s001.tiff]
