# Supplementary material for: Human Parvovirus B19 Induced Apoptotic Bodies Contain Altered Self-Antigens that are Phagocytosed by Antigen Presenting Cells
Source: PLoS One. 2013 Jun 12;8(6):e67179. doi: 10.1371/journal.pone.0067179 (PMC3680405; doi:10.1371/journal.pone.0067179)
Supplement: Table S1 — Purified ApoBods in consequence of NS1 expression presented high quantity and green signal. Quantity of purified ApoBods from transduced cells with AcEGFP and AcEGFP-NS1, and treated with staurosporine control from FC 3 different assays were analyzed. The results from each condition presented as mean ± SEM (N = 3). P value < 0.05 is significantly; *compare between AcEGFP and AcEGFP-NS1, **compare between AcEGFP and staurosporine, and ***compare between AcEGFP-NS1 and staurosporine. [file pone.0067179.s002.docx]

**Table S1.**

| **Apoptotic bodies  (ApoBods)** | Quantity/min  (mean ± SEM) | Green signal % from 10,000 events  (mean ± SEM) | Minute to collect 10,000 events  (mean ± SEM) |
| --- | --- | --- | --- |
| ***Ac*EGFP transduction** | 430 ± 50.74 | 18.06 ± 2.44 | 6.47 ± 0.29 |
| ***Ac*EGFP-NS1 transduction** | 910 ± 70.00 * | 31.77 ± 4.74 *,*** | 2.57 ± 0.23 * |
| **Staurosporine** | 995 ± 70.00 ** | 8.44 ± 1.32 | 2.50 ± 0.29 ** |
